# Graph-Driven Micro-Expression Rendering with Emotionally Diverse Expressions for Lifelike Digital Humans

**DOI:** 10.3390/biomimetics10090587

**Published:** 2025-09-03

**Authors:** Lei Fang, Fan Yang, Yichen Lin, Jing Zhang, Mincheol Whang

**Affiliations:** 1 Department of Emotion Engineering, Sangmyung University, Seoul 03016, Republic of Korea; flengel1983@gmail.com (L.F.); youngfan062@gmail.com (F.Y.); 2 Department of Building Engineering, Hebei Vocational University of Industry and Technology, Shijiazhuang 050091, China; linyichen775@gmail.com; 3 Jingjinji Spatial Intelligent Perception Collaborative Innovation Center, Hebei University of Engineering, Handan 056009, China; zhangjing01@hebeu.edu.cn; 4 Department of Human-Centered Artificial Intelligence, Sangmyung University, Seoul 03016, Republic of Korea

**Keywords:** micro-expressions, FACIAL Action Coding System (FACS), graph convolutional network (GCN), action units, spatiotemporal feature extraction, digital humans

## Abstract

Micro-expressions, characterized by brief and subtle facial muscle movements, are essential for conveying nuanced emotions in digital humans, yet existing rendering techniques often produce rigid or emotionally monotonous animations due to the inadequate modeling of temporal dynamics and action unit interdependencies. This paper proposes a graph-driven framework for micro-expression rendering that generates emotionally diverse and lifelike expressions. We employ a 3D-ResNet-18 backbone network to perform joint spatio-temporal feature extraction from facial video sequences, enhancing sensitivity to transient motion cues. Action units (AUs) are modeled as nodes in a symmetric graph, with edge weights derived from empirical co-occurrence probabilities and processed via a graph convolutional network to capture structural dependencies and symmetric interactions. This symmetry is justified by the inherent bilateral nature of human facial anatomy, where AU relationships are based on co-occurrence and facial anatomy analysis (as per the FACS), which are typically undirected and symmetric. Human faces are symmetric, and such relationships align with the design of classic spectral GCNs for undirected graphs, assuming that adjacency matrices are symmetric to model non-directional co-occurrences effectively. Predicted AU activations and timestamps are interpolated into continuous motion curves using B-spline functions and mapped to skeletal controls within a real-time animation pipeline (Unreal Engine). Experiments on the CASME II dataset demonstrate superior performance, achieving an F1-score of 77.93% and an accuracy of 84.80% (*k*-fold cross-validation, k = 5), outperforming baselines in temporal segmentation. Subjective evaluations confirm that the rendered digital human exhibits improvements in perceptual clarity, naturalness, and realism. This approach bridges micro-expression recognition and high-fidelity facial animation, enabling more expressive virtual interactions through curve extraction from AU values and timestamps.

## 1. Introduction

The increasing use of virtual humans in education, healthcare, cultural communication, and immersive interaction has established emotional expressiveness as a key metric for assessing interaction quality and user experience. Beyond basic expression mapping and emotion classification, real-world applications require virtual agents to convey emotions with greater granularity, diversity, and dynamic intensity—fostering trust, empathy, and emotional resonance [1,2].

Micro-expressions, brief (1/25 to 1/5 s) involuntary facial muscle movements, are critical nonverbal cues for identifying concealed or suppressed emotions [3]. Their transient and involuntary nature makes them highly effective in revealing subtle emotional states. Recent studies have shown that such fine-grained signals are essential for affective evaluation in multimodal systems—including EEG, eye-tracking, and virtual reality [4,5]—highlighting their value in enhancing the perceptual realism of emotionally responsive virtual agents.

However, most existing virtual human systems rely on macro-expression-driven skeletal templates [3], which are insufficient for capturing nuanced facial dynamics or rapid emotional transitions. These limitations result in coarse expression granularity, reduced variation, and restricted emotional range, impairing the system’s ability to render subtle affective changes—especially in high-fidelity interaction scenarios.

Recent advances in virtual human emotion synthesis have leveraged generative diffusion frameworks and Transformer-based sequence models to improve the photorealism and controllability of facial animation. For example, EMO produces highly expressive talking-head videos from audio via an Audio2Video diffusion pipeline [6]; AnimateMe formulates diffusion directly on facial meshes to synthesize controllable 4D facial expressions [7]; and FaceFormer shows that Transformer decoders capture long-term temporal dependencies for realistic 3D facial animation [8]. However, these pipelines rarely address micro-expressions, which require explicit modeling of action unit (AU) dependencies and micro-level onset/apex/offset timing.

Importantly, micro-expressions cannot be reproduced by simply superimposing high-frequency blendshapes on top of macro-expression rigs. Linear blendshape interpolation assumes the independent, time-invariant superposition of local controls, which often yields implausible or noisy dynamics for subtle deformations [7]. In contrast, micro-expressions involve the temporally synchronized co-activation of multiple AUs, exhibiting a physiologically plausible onset-peak-offset pattern [9]. Ignoring such dependencies causes phase inconsistency, implausible co-contractions, and the loss of subtle intensity transitions—precisely the cues that observers use to perceive micro-expressions.

In the domain of micro-expression research, recognition and generation are typically addressed as separate tasks, lacking an integrated pipeline that connects video-based emotion perception, structural modeling, and animation synthesis. Moreover, few methods account for the structured dependencies among action units (AUs, standardized descriptors of facial muscle movements) or incorporate continuous emotion intensity modulation. This fragmented architecture reduces the responsiveness, realism, and expressive depth of virtual humans in dynamic, context-aware settings.

To tackle this challenge, we propose a unified framework for micro-expression modeling and 3D animation, enabling end-to-end synthesis of expressive facial behavior without explicit emotion classification. All components prioritize precise action unit (AU) extraction to achieve realistic rendering. Specifically, we integrate a symmetric AU graph with a B-spline curve mapper to encode co-activation probabilities—implemented as a post-processing step via an Unreal Engine 5 plugin to preserve algorithmic performance—maintaining continuous onset, apex, and offset timing for controlled micro-expression synthesis. The framework comprises three core components: (1) Spatiotemporal Feature Extraction: A 3D-ResNet-18 network extracts motion-aware features from emotion-rich video sequences, identifying onset, apex, and offset frames of micro-expressions to derive precise timestamps for curve generation. (2) Structural Dependency Modeling: A graph convolutional network (GCN) leverages an undirected, symmetric adjacency matrix based on AU co-occurrence probabilities to capture interdependent facial activations for accurate AU value extraction. (3) 3D Animation Synthesis: Extracted timestamps and AU values are converted into continuous animation control curves using a lookup table. B-spline interpolation, implemented via C++ in Unreal Engine 5 (as detailed in Section 3.3), ensures smooth transitions without impacting upstream feature extraction or graph modeling, driving the facial rig in Unreal Engine to produce fine-grained, high-fidelity facial animations.

To validate the framework, we conducted a perceptual study evaluating the synthesized facial expressions across three dimensions: clarity, naturalness, and authenticity. Results demonstrate that our system significantly improves emotional recognizability and visual realism, particularly in distinguishing similar negative emotions such as fear and disgust.

In summary, this work presents an integrated framework that combines micro-expression recognition, AU-based structural modeling, and high-resolution animation synthesis. By addressing both temporal localization and structural dependencies, the system advances controllable, emotionally differentiated, and perceptually realistic facial animation for virtual humans, offering a scalable solution for affective computing and immersive human–machine interaction.

## 2. Related Work

### 2.1. Trends in Micro-Expression Recognition

Micro-expressions—brief facial movements lasting less than 0.5 s [10] with low intensity—remain a major challenge in visual behavior analysis. Early research relied on handcrafted descriptors such as Local Binary Patterns on Three Orthogonal Planes (LBP-TOPs) [11], Histogram of Oriented Optical Flow (HOOF) [12], and traditional optical flow methods [13,14]. While these techniques yielded reasonable performance in controlled settings (e.g., CASME II [15]), they exhibit limited robustness to illumination variation, head motion, and dynamic interactions among facial action units (AUs), restricting their applicability in real-world scenarios.All these methods, including our proposed framework, are oriented toward serving micro-expression curve extraction, focusing on timestamps and AU values without involving emotion classification, to facilitate mapping to digital human expressions via a lookup table.

With the emergence of deep learning, micro-expression recognition has made significant strides. Convolutional 3D networks (3D-CNNs), first proposed by Tran et al. [16], enabled the joint modeling of spatial and temporal cues and have been adapted for micro-expression recognition [17,18]. Recurrent Neural Networks (RNNs) and Long Short-Term Memory (LSTM) models [19,20] improved the modeling of temporal dependencies between facial regions, facilitating the learning of dynamic AU relationships. More recently, graph convolutional networks (GCNs), originally introduced by Kipf and Welling [21], have been applied to model AU co-activation structures [22,23], demonstrating improved robustness for subtle expression recognition.

Graph convolutional networks (GCNs) excel in modeling facial action units (AUs) by capturing structural dependencies and asymmetric interactions through graph propagation, improving robustness to subtle micro-expression variations compared to traditional convolutional methods, which treat features independently. Using adjacency matrices based on AU co-occurrence probabilities, GCNs propagate contextual information, enhancing generalization across subjects and resilience to noise in low-amplitude signals. However, their performance hinges on the quality of the predefined graph structure, which can introduce bias if derived from limited or imbalanced datasets. Additionally, GCNs face challenges with computational complexity as graph size increases, limiting real-time use in resource-constrained settings, and deeper layers may over-smooth features, reducing the detail needed for micro-expression analysis.Foundational works, such as C3D for joint spatiotemporal learning [16] and graph convolutional networks for inference on graph-structured data [21], form the basis of many contemporary micro-expression recognition (MER) pipelines.

End-to-end convolutional approaches have also been explored. Wang et al. [24] utilized a combination of a 2D-CNN and a 1D-CNN to extract spatial and temporal features, refining temporal localization; Zhang et al. [18] enhanced temporal proposal accuracy; Yap et al. [17] adopted 3D-CNNs for joint spatiotemporal modeling; and Leng et al. [25] introduced Boundary-Sensitive Networks (BSNs) to accommodate blended macro- and micro-expressions. Nevertheless, most existing methods still focus on basic emotion categories and fail to adequately model complex emotional states, subtle facial variations, and inter-individual differences.

Traditional animation pipelines typically rely on static blendshape interpolation, which cannot capture the nuanced dynamics of AU interactions or represent the diversity of human emotional expressions. Recent generative architectures, such as diffusion models and Transformers, have enhanced the realism of macro-expression synthesis; however, micro-expression modeling remains inadequately addressed in these frameworks. For instance, Lei et al [26] proposed a vision Transformer variant that captures long-range dependencies in micro-expression sequences, achieving enhanced robustness to noise. Despite this progress, despite these advances, most methods overlook the asymmetric interdependencies between AUs, whereas our framework addresses this through a directed graph structure. Diffusion models have also emerged for generative tasks, but their application to micro-expression synthesis remains limited due to high computational demands.

To bridge this gap, we propose a unified framework that integrates micro-expression recognition and synthesis using AU-guided graph modeling. Specifically, we construct directed graphs based on AU co-occurrence probabilities to represent AU dependencies, and combine GCN-based structural inference with temporal feature encoding. This enables the generation of diverse and temporally aligned emotional trajectories. The proposed approach addresses the limitations of prior work in capturing fine-grained emotional dynamics and provides a robust pathway for high-realism and expressive facial animation in virtual humans.

### 2.2. AU Detection

Action units (AUs), the fundamental components of the Facial Action Coding System (FACS) [27], represent subtle facial muscle movements and provide a physiologically grounded framework for fine-grained expression analysis. Relative to direct classification of discrete emotions, AU detection focuses on specific muscle activations, enhancing interpretability while mitigating annotation biases. This structured approach underpins micro-expression recognition and affective modeling.

Early AU detection methods relied on static appearance features, such as facial textures and keypoints, but struggled to capture temporal dynamics and nonlinear co-activation patterns among multiple AUs—particularly in brief micro-expressions involving intricate muscular interactions. Deep learning has markedly advanced AU detection, with models incorporating convolutional neural networks (CNNs), Long Short-Term Memory (LSTM) networks, and graph convolutional networks (GCNs) excelling in discerning subtle muscle dynamics.

For example, Liu et al. [28] modeled AU co-activation relationships using GCNs, enhancing both detection robustness and cross-dataset generalization.Graph convolutional networks (GCNs) have been employed for action unit (AU) detection, effectively modeling AU co-activations to enhance robustness on the BP4D-Spontaneous and DISFA datasets [28].

Despite these advances, three persistent challenges remain. First, the scarcity of large-scale, high-quality AU-labeled datasets hampers generalizability. Second, many methods assume static AU relationships, overlooking the time-varying yet symmetric interactions that define spontaneous micro-expressions, rooted in anatomical bilateralism. Third, existing approaches often lack a direct mapping from AU detection to animation control, complicating the synthesis of emotionally diverse and expressive behaviors.

### 2.3. Micro-Expression Modeling in Virtual Humans

Micro-expression modeling aims to transform recognized subtle facial signals into a stream of animation control parameters. The core challenges lie in ensuring temporal accuracy, dynamic naturalness, and controllability within 3D animation pipelines.

Several representative systems—such as SARA [29] and ARIA [30]—have explored expressiveness and interactivity to some extent. However, they suffer from limited control over fine-grained facial details. For instance, SARA utilizes Behavior Markup Language (BML) to control facial states and supports the synthesis of basic emotions. Nevertheless, it lacks granularity in modeling subtle muscular transitions and exhibits rigidity in facial expression transitions. The ARIA platform adopts a modular design that separates input processing, agent generation, and animation output. Despite this structured architecture, its output still heavily depends on predefined motion templates, making it difficult to accurately reproduce realistic micro-facial dynamics. To compare with recent virtual human pipelines, EMO (audio-driven expressive portrait video) [6], AnimateMe (mesh-space diffusion for 4D facial animation) [7], and FaceFormer (speech-driven 3D facial animation) [8] enhance photorealism and controllability but do not explicitly model action unit-level micro-dynamics. Coface [31] Consequently, despite these efforts, micro-expression modeling for virtual humans remains underexplored, particularly regarding AU-structured control and micro-timing preservation.

Moreover, while recent advances in animation generation have benefited from large-scale algorithmic models, the physical realism of micro-expression modeling remains an underexplored area, requiring further investigation.

### 2.4. Comparative Analysis and Methodological Innovations

Despite notable progress in micro-expression recognition, most existing methods focus on either temporal localization or emotion classification, without offering a unified mechanism to directly integrate with virtual human facial animation systems. Moreover, current animation pipelines typically rely on static expression templates or pre-defined blendshapes, which are inadequate for modeling the dynamic interplay and nuanced transitions among facial action units (AUs), often resulting in rigid and emotionally limited behaviors. Our approach differs by (i) learning an undirected symmetric AU graph that encodes co-activation probabilities, and (ii) mapping predictions to continuous B-spline curves—generated via the Unreal Engine 5 plugin without affecting core algorithmic performance—that preserve onset/apex/offset timing, thereby enabling controllable yet physiologically plausible micro-expressions in real time, all serving AU-based curve extraction for emotional diversity without explicit classification.

Based on the analysis of Table 1, handcrafted methods such as LBP-TOP [11] and HOOF [12] exhibit robustness to illumination variations or effective motion capture capabilities; however, they are constrained by their reliance on static features or susceptibility to noise. Deep learning approaches, including CNN + LSTM [32] and MER-GCN [33], demonstrate proficiency in modeling sequential dependencies or capturing AU co-occurrences, yet they overlook structural relationships or lack emotion modulation. Animation systems like SARA [29] and ARIA [30] support emotion synthesis or offer flexible architectures, but they are limited by the absence of micro-expression transitions or their dependence on predefined templates. In contrast, the proposed AU_GCN_CUR method integrates 3D-ResNet with spatiotemporal AU modeling and end-to-end animation, significantly enhancing emotional diversity, albeit at the cost of increased computational complexity.

#### 2.4.1. Methodological Innovations

To address these limitations, this study proposes a unified, closed-loop framework that links micro-expression recognition, temporal segmentation, and facial animation control into an end-to-end process. This end-to-end system facilitates the seamless integration of AU-based emotion recognition, temporal segmentation, and 3D facial animation synthesis. The proposed framework introduces three core innovations:

- Graph-Based Modeling of Action Units: Each AU is represented as a node in a symmetric adjacency matrix, capturing inherent facial muscle dependencies. A graph convolutional network (GCN), built on AU co-occurrence statistics, enhances structural sensitivity and generalization for precise AU value extraction, driving animation curve generation for realistic digital human expressions without emotion classification.
- Joint Spatiotemporal Feature Extraction: To simultaneously capture spatial configurations and temporal dynamics, a 3D-ResNet-18 backbone is adopted. To enhance the modeling of subtle temporal variations, the backbone is further integrated with an Enhanced Long-term Recurrent Convolutional Network (ELRCN), thereby improving the sensitivity to transient and low-intensity motion cues, which are critical for micro-expression analysis.
- Emotion-Driven Animation Mapping Mechanism: The extracted AU activation patterns are mapped into parameterized facial muscle trajectories via continuous motion curves, which in turn drive the expression synthesis module of the virtual human. This mapping strategy enables the generation of contextually appropriate and emotionally expressive facial animations, exceeding the expressive capacity of traditional template-based methods.

In summary, this work marks a significant step forward in bridging micro-expression recognition and high-fidelity animation synthesis, paving the way for emotionally responsive and behaviorally coherent virtual human systems. It not only enhances recognition accuracy and temporal precision but also establishes a controllable, closed-loop animation generation paradigm grounded in fine-grained AU dynamics.

#### 2.4.2. Architectural Innovations

In recent years, many studies have employed CNNs to extract spatial features from individual frames, while incorporating Long Short-Term Memory (LSTM) or Recurrent Neural Network (RNN) structures to model temporal dependencies across frames [35], thereby capturing the dynamic evolution of facial movements. Others have adopted feature aggregation and encoding strategies such as bilinear models, VLAD, and Fisher encoding [16,30]. These networks commonly use three-dimensional convolutional and pooling kernels, extending conventional 2D spatial operations into the temporal dimension *t*, to directly model spatiotemporal features in video sequences.

For example, in optical flow-based CNNs, the temporal kernel size *d* is often set to 10. Tran et al. [16] explored 3D CNNs with kernel sizes of 3×3×3 and extended the ResNet architecture using 3D convolutions. Feichtenhofer et al. [36] further proposed a 3D spatiotemporal pooling strategy. Sun et al. [37] decomposed 3D convolutions into 2D spatial and 1D temporal convolutions to reduce computational complexity while preserving modeling capacity. Carreira et al. [38] proposed inflating a pre-trained 2D Inception-V1 architecture into a 3D one by extending all filters and pooling kernels along the temporal dimension *d*.

However, in practical applications, the varying number of frames across video sequences poses challenges for direct temporal modeling. To address alignment issues, time normalization techniques are often employed to produce fixed-length sequences. Pfister et al. [39] introduced a popular normalization algorithm—the temporal interpolation model (TIM)—which maps video frames along a time-constrained manifold. At the feature level, frame-wise features are aggregated to form a unified representation.

Inspired by these studies, our proposed system adopts a two-stage architecture for micro-expression analysis:

- Spatiotemporal Feature Extraction: Given that micro-expressions (MEs) are brief (<0.5 s) and subtle in amplitude, we utilize a lightweight yet effective backbone—3D-ResNet-18—for end-to-end modeling of video segments. The 3D convolutional neural network (3D-CNN) slides jointly across spatial (*x*, *y*) and temporal (*t*) dimensions, enabling the network to perceive fine-grained motion variations between consecutive frames. This makes it particularly suitable for temporally sensitive and low-amplitude signals such as MEs [16]. Additionally, the Enhanced Long-term Recurrent Convolutional Network (ELRCN) [40] incorporates two learning modules to strengthen both spatial and temporal representations.
- AU Relationship Modeling: AUs, as defined in the Facial Action Coding System (FACS), are physiologically interpretable units that encode facial muscle movements and exhibit cross-subject consistency. Therefore, they are widely used in micro-expression analysis and synthesis. Liu et al. [28] manually defined 13 facial regions and used 3D filters to perform convolution over feature maps for AU localization. Inspired by this approach, we introduce a GCN to model co-occurrence relationships between AUs. Each AU is represented as a node in a graph, and the edge weights are defined based on empirical co-occurrence probabilities.

## 3. Framework and Methods

### 3.1. Overall System Architecture

The proposed framework introduces a closed-loop pipeline for micro-expression localization and animation synthesis in virtual humans, overcoming the limitations of conventional coarse-grained templates that neglect subtle dynamics and fail to integrate with 3D rendering engines.

**Figure 1:**
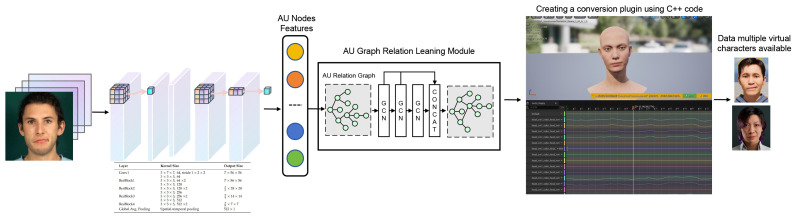
The framework leverages 3D-ResNet-18 to extract spatiotemporal AU features, employs dual regression for timestamp prediction, and utilizes AU-GCN-CUR to capture dependencies, enabling efficient micro-expression localization and realistic facial animation synthesis in Unreal Engine.

As illustrated in Figure 1, a 3D-ResNet-18 backbone extracts spatiotemporal features from input sequences. These features drive dual regression branches to predict normalized onset and offset timestamps in [0, 1], optimized by MSE loss with a temporal order constraint for consistency.Meanwhile, the human face exhibits symmetry, and AU relationships, grounded in co-occurrence and facial anatomy, are typically considered symmetric and non-directional. The proposed system processes these relationships through the AU-GCN-CUR module to capture inter-AU dependencies and generate structured representations. End-to-end training integrates losses for localization and AU embedding.

In inference, normalized timestamps $\left({\hat{y}}_{\mathrm{start}},{\hat{y}}_{\mathrm{end}}\right)$ are mapped to frame indices, while AU predictions generate continuous control curves for real-time facial actuation in Unreal Engine. This unified design enables precise synthesis across intensities, surpassing static templates in flexibility and fidelity.

On CASME II  [15], where sequences are preprocessed to a fixed length, the framework outperforms baseline algorithms on this dataset, with subjective evaluations confirming enhanced clarity and authenticity.

### 3.2. Temporal Segmentation

**Figure 2:**
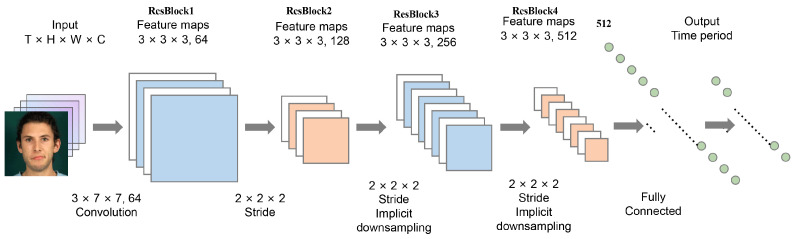
This figure illustrates a lightweight convolutional neural network architecture based on 3D-ResNet-18, which employs 3 × 3 × 3 convolutional kernels to extract spatiotemporal features and, combined with global average pooling, generates a 512-dimensional feature vector, further enhanced by temporal interpolation and regression branches for precise micro-expression localization.

To address challenges in micro-expression recognition, as shown in Figure 2, where subtle and brief facial dynamics elude single-frame capture, we employ a 3D convolutional neural network (3D-CNN) for unified spatiotemporal modeling of video sequences. A lightweight 3D-ResNet-18 architecture [41] serves as the backbone, extracting features across spatial (x,y) and temporal (*T*) dimensions via 3D kernels to enhance sensitivity to fine-grained variations. This prioritizes temporal dynamics over spatial-only approaches, as in MotionSC [42], while integrating explicit AU relational modeling that is absent in the latter.

Input sequences are preprocessed to a fixed length (e.g., 16 frames using the temporal interpolation model, TIM) and processed with 3×3×3 kernels at stride 1 to detect transient dynamics. Output is a 512-dimensional feature vector $F\in R^{512}$ post global average pooling (GAP), retaining spatiotemporal action patterns.

Temporal segmentation appends two regression branches to predict normalized onset and offset timestamps in [0, 1]:

(1) $$\left({\hat{y}}_{\mathrm{start}},{\hat{y}}_{\mathrm{end}}\right)=\mathrm{Linear}\left(F\right)$$

where ${\hat{y}}_{\mathrm{start}}$ and ${\hat{y}}_{\mathrm{end}}$ are the predicted normalized onset and offset timestamps, respectively; $F\in R^{512}$ is the 512-dimensional spatiotemporal feature vector extracted from the 3D-ResNet-18 backbone after global average pooling (GAP); and Linear(·) denotes a fully connected linear layer. The optimization employs a combined loss function:

(2) $$L_{seg}=L_{cls}+\lambda_{1}L_{reg}+\lambda_{2}L_{order}$$

where $L_{cls}=-\sum_{i=1}^{N}y_{i}\log\left({\hat{y}}_{i}\right)$ is the classification loss, with $y_{i}$ and ${\hat{y}}_{i}$ denoting the ground-truth and predicted classification labels (e.g., micro-expression categories such as happiness or surprise) for the *i*-th sample, and *N* is the number of samples; $L_{reg}=\sum_{i=1}^{N}\left[{\left(t_{\mathrm{start},i}-{\hat{t}}_{\mathrm{start},i}\right)}^{2}+{\left(t_{\mathrm{end},i}-{\hat{t}}_{\mathrm{end},i}\right)}^{2}\right]$ is the regression loss, with $t_{\mathrm{start},i}$ and $t_{\mathrm{end},i}$ as the ground-truth normalized onset/offset timestamps, and ${\hat{t}}_{\mathrm{start},i}$ and ${\hat{t}}_{\mathrm{end},i}$ as the predictions; $L_{order}=\sum_{i=1}^{N}\max\left(0,{\hat{t}}_{\mathrm{start},i}-{\hat{t}}_{\mathrm{end},i}\right)$ is the order constraint to ensure onset precedes offset; and $\lambda_{1}=0.5$, $\lambda_{2}=0.1$ are hyperparameters to balance the losses and enforce temporal order consistency. This loss function performs multi-task learning, simultaneously regressing the normalized onset (${\hat{y}}_{\mathrm{start}}$) and offset (${\hat{y}}_{\mathrm{end}}$) timestamps while classifying the micro-expression category (or existence). The order constraint ensures temporal consistency by penalizing cases where ${\hat{y}}_{\mathrm{start}}>{\hat{y}}_{\mathrm{end}}$.

Architecture details appear in Table 2, illustrating progressive temporal downsampling for contextual capture.

**Figure 3:**
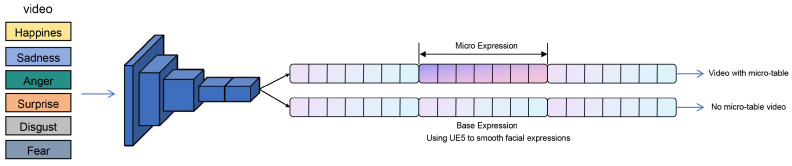
Overall two-stage architecture and tensor shapes. Stage I: A 3D-ResNet-18 backbone extracts joint spatiotemporal features from video clips with progressive temporal downsampling; kernel/stride per block follows Table 1 (temporal strides annotated on each block). Stage II: AU features feed into the graph-based module and the spline-based curve mapper for real-time facial animation. Tensor sizes (T, H, W, C) are shown at major nodes.

AU features from *F* form nodes in directed graph G=(V,E), where *V* denotes AU nodes (e.g., 19 in CASME II) initialized as one-hot vectors, and as illustrated in Figure 3, *E* edges via co-activation probabilities $A_{ij}=P\left(U_{i}|U_{j}\right)=\frac{N_{i\cap j}}{N_{j}}$, thereby generating a symmetric adjacency matrix. An emotional layer augments nodes (e.g., 7D vector for categories) to distinguish states like subtle joy vs. polite surprise. Features refine via GCN propagation:

(3) $$H^{\left(l\right)}=\sigma \left(AH^{\left(l-1\right)}W^{\left(l-1\right)}\right)$$

where $H^{\left(l\right)}$ is the feature matrix at layer *l*; *A* is the asymmetric adjacency matrix derived from AU co-occurrence probabilities; $W^{\left(l-1\right)}$ is the learnable weight matrix at layer l−1; and σ(·) is the activation function with loss $L_{graph}=\alpha L_{AU}+\left(1-\alpha \right)L_{emotion}$ (α=0.7), $L_{AU}$ binary cross-entropy for multi-label AU classification, and $L_{emotion}$ cross-entropy for emotions. This graph method captures inter-AU dependencies, surpassing MotionSC’s temporal 3D convolutions.

### 3.3. Graph Structure Modeling Based on AU Co-Occurrence Relationships

To model co-activation patterns and symmetries, action units (AUs) are represented as nodes in an undirected graph. AU features are extracted from the 3D ConvNet output. The graph is G=(V,E), where *V* comprises AU nodes, each with a 512-dimensional spatiotemporal feature vector; *E* uses co-occurrence probabilities:

(4) $$A_{ij}=\frac{N_{ij}+N_{ji}}{N_{i}+N_{j}}$$

where $A_{ij}$ is the element of the adjacency matrix $A\in R^{n\times n}$; $U_{i}$ and $U_{j}$ represent the *i*-th and *j*-th action units; $N_{ij}$ is the co-occurrence count of AUs *i* and *j*, yielding a symmetric adjacency matrix $A\in R^{n\times n}$, with $N_{ij}$ the co-occurrence count of AUs *i* and *j*, and $N_{j}$ the total count of AU *j*. This symmetric design is justified by the bilateral symmetry of human faces, where AU relationships are based on co-occurrence and facial anatomy analysis (as per the FACS [26]), which are typically undirected and symmetric (e.g., AU12 and AU6 co-activations in happiness are bidirectional). Such relationships align with the design of classic spectral GCNs for undirected graphs, assuming that adjacency matrices are symmetric to model non-directional co-occurrences effectively.

Graph convolutions refine AU intensity predictions, using 2–3 layers:

(5) $$H^{\left(l+1\right)}=\sigma \left({\tilde{D}}^{-1/2}\tilde{A}{\tilde{D}}^{-1/2}H^{\left(l\right)}W^{\left(l\right)}\right)$$

where $\tilde{A}=A+I$, $\tilde{D}$ is the degree matrix, $W^{\left(l\right)}$ are the weights, and σ is ReLU. A self-attention pooling layer retains key nodes (ratio p=0.5) by scoring *Z*, selecting top-*k*, and updating the features/matrix. The overall model processes an input video to extract the onset and offset timestamps of micro-expressions, along with the corresponding AU node values. These AU values are then mapped against an AU-to-rig control table (e.g., as in Table 3) to enable the reconstruction and rendering of both micro-expressions and basic expressions on digital human models. All algorithmic components, including spatiotemporal feature extraction and graph-based modeling, are designed to serve the precise extraction of facial expressions, ensuring accurate temporal localization and AU activation for lifelike animation synthesis.

### 3.4. Animation Synthesis with Diverse Emotional Profiles

**Figure 4:**
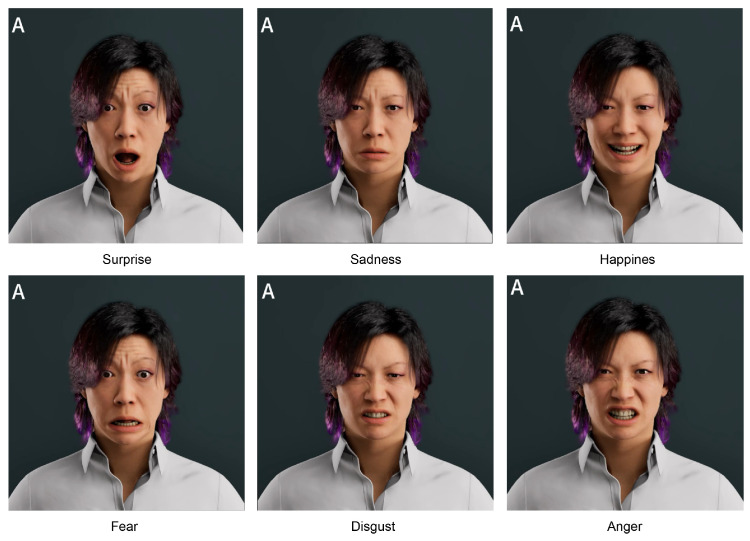
Generated facial expressions with micro-expression modulation. Six basic emotions (Surprise, Sadness, Happiness, Fear, Disgust, Anger) rendered by the proposed AU-to-curve mapping in Unreal Engine.

The predicted AU signals are transformed into smooth animation curves via cubic spline interpolation, modulated by emotion-specific intensity profiles, and mapped to rig controls in a commercial engine (e.g., Unreal Engine [43]) to drive lifelike digital human expressions. As illustrated in Figure 4, this process not only captures micro-movements but also encodes differentiable emotional signatures, such as restrained fear or mild disgust.

For each AU signal sequence s(t), a continuous curve is generated using cubic B-spline interpolation:

(6) $$c\left(t\right)=\sum_{i=0}^{3}b_{i}B_{i}\left(t\right)$$

where c(t) is the interpolated animation curve at time *t*; $b_{i}$ are the control points derived from AU activations and displacements; and $B_{i}\left(t\right)$ are the cubic B-spline basis functions.

The curve is then modulated as follows:

(7) $$c^{\prime }\left(t\right)=c\left(t\right)\cdot m_{e}\cdot i_{v}$$

where $m_{e}$ is the emotion-specific modulator (e.g., high for joy, low for fear); and $i_{v}\in \left[0,1\right]$ represents intensity variation, sampled from user input or dataset distributions to preserve physiological realism in AU interrelations, as informed by the AU-GACN’s intensity control. In Unreal Engine, the refined curves are dynamically generated using the Curve Editor  API:

- Invoke the FindRow method to match the input expression curve with entries in the AU dictionary and extract the corresponding RowValue (a time–displacement key-value pair).
- Based on RowValue->Time and RowValue->Disp, create animation keyframes via FKeyHandle and append them to the animation curve.
- Use SetKeyTangentMode to set the tangent mode to automatic (RCTMAuto), and call SetKeyInterpMode to set the interpolation mode to cubic (RCIMCubic), improving transition quality between keyframes.

The final curve is saved into the Unreal Engine project, ensuring consistency between the generated expressions and the imported animation data. This enables the precise control and real-time preview of facial animation. The representative AU–to–rig mapping used in Unreal is summarized in Table 3. For specific mapping details, please refer to Table S1 in the Supplementary Materials.

The mapping step, implemented through a custom C++ plugin, processes AU signals to animation curves with an average latency of approximately 10–20 ms per frame (tested on a Colorful Co., Ltd. NVIDIA RTX 3080 GPU (Shenzhen, Guangdong, China) with an Intel Core i7-11700K CPU (Intel Corporation, Santa Clara, CA, USA)), enabling real-time performance at 30–60 FPS. This low latency is achieved by optimizing the B-spline interpolation and Curve Editor API calls within the plugin, ensuring seamless integration without frame drops in dynamic scenarios.

## 4. Experiments

To evaluate the effectiveness of the proposed micro-expression recognition and generation system, a dual-validation framework is adopted, encompassing both objective metrics and subjective user experience.

On the objective side, we assess the temporal prediction accuracy and overall model performance through cross-validation experiments. On the subjective side, user feedback is collected via questionnaire-based evaluations, in which participants rate the generated virtual human animations across three perceptual dimensions: clarity, naturalness, and authenticity.

### 4.1. System Performance Evaluation

#### 4.1.1. Dataset and Experimental Settings

We evaluated our framework using the CASME II dataset [15], a widely adopted benchmark for micro-expression analysis, comprising 247 video sequences from 26 subjects. These sequences are annotated with onset/offset frames and emotion labels, captured at 200 FPS under controlled lighting. The dataset’s high temporal resolution and subtle expression variations pose a challenging testbed. Our proposed pipeline processes facial videos to generate emotion-differentiated micro-expression animations for 3D digital humans. The framework consists of three core stages: the temporal segmentation of micro-expressions, emotion-labeled action unit (AU) relationship modeling, and real-time animation curve mapping with variable emotional intensity. Temporal segmentation employs a 3D ConvNet to detect onset and offset frames of micro-expressions. Extracted AUs are represented as nodes in a directed graph with emotion labels, capturing co-activation patterns and asymmetries. A graph convolutional module propagates contextual information to refine AU intensity predictions and distinguish emotional states (e.g., subtle joy versus polite surprise). The end-to-end design achieves real-time performance (30–60 FPS in experiments, hardware-dependent).

#### 4.1.2. Implementation Details

In this study, we adopt an 18-layer 3D-ResNet (ResNet3D-18) as the backbone network for spatiotemporal feature extraction from micro-expression sequences. Given the limited number of training samples, we employ sample-level updates in each training iteration, using a single video sequence as input to enhance adaptability to small-sample scenarios.

As shown in Table 2, the model consists of four residual blocks. The input sequence has a temporal length of *T*, and each frame is resized to a spatial resolution of 112×112 and normalized. This preprocessing is consistently applied to all training and validation data. In the 3D convolutional layers, the network contains five residual modules, each responsible for progressively extracting coarse-to-fine spatiotemporal features. Each residual block comprises two 3D convolutional layers with kernel sizes of 3×3×3, followed by ReLU activation and batch normalization. Global average pooling is used to downsample the output feature map to a size of 512×1, with spatial stride control while keeping the temporal dimension intact.

The GCN is configured with two stacked layers. The input is a one-hot encoding of 12 AU nodes, resulting in an initial feature matrix of size 12×d, where *d* is the feature dimension. The adjacency matrix *A* is constructed in a data-driven manner, with each element $A_{ij}$ defined as the conditional probability $P(U_{i}|U_{j})$. The output dimensions of the two GCN layers are set to 1024 and 512, respectively.

The CASME II dataset [15] is partitioned using Leave-One-Subject-Out (LOSO) and *k*-fold cross-validation. In *k*-fold validation, the dataset is divided into *k* subsets, with one subset used for validation and the remaining for training in each fold. Both strategies help to prevent the model from overfitting to specific data partitions and enhance generalization.

For model validation, we employ two cross-validation strategies: Leave-One-Subject-Out (LOSO) and 5-fold cross-validation. In LOSO, data from one subject are excluded for validation in each iteration to mitigate subject-specific biases, which are prevalent in micro-expression datasets due to inter-individual variations in facial dynamics. In 5-fold validation, the dataset is partitioned into five equal subsets, with each fold using one subset for validation and the others for training. These strategies ensure robust generalization and prevent overfitting to specific data partitions. All experiments were conducted on the CASME II dataset [15], with preprocessing including frame normalization to 112 × 112 resolution and temporal interpolation to a fixed length of 16 frames.

As shown in Table 4, in the *k*-fold cross-validation setting, the 3D-ResNet model achieved an average accuracy of 84.87% (±3.80 std. dev. across folds), demonstrating a substantial improvement over the pre-trained model, which reached 61.92% (±6.53 std. dev. across folds). This suggests that, under training strategies involving moderate data volumes and relatively balanced sample distributions, features learned from scratch are better equipped to capture the subtle variations that are inherent in micro-expressions. In the LOSO validation setting, the 3D-ResNet model again outperformed its pre-trained counterpart, attaining an average accuracy of 80.51% (±6.58 std. dev. per subject) compared to 56.20% (±28.31 std. dev. per subject). These findings indicate that pre-trained models encounter difficulties when transferring to the micro-expression domain, primarily due to semantic domain discrepancies.

To ensure experimental reproducibility, all training was performed on hardware equipped with an NVIDIA RTX 3090 GPU (24 GB VRAM), an Intel Core i9-12900K CPU, and 64 GB of RAM, using the PyTorch 2.0 framework. The random seed was set to 42 for initializing network weights and data shuffling.

Table 5 presents a comparison of accuracy (%) for baseline models based on *k*-fold and LOSO evaluations. As part of the baseline comparison, ThreeDFlow, CNN + LSTM, and CapsuleNet achieved accuracies of 63.48%, 63.78%, and 44.83%, respectively in *k*-fold evaluation, which dropped to 42.58%, 48.35%, and 31.16% in LOSO evaluation. In contrast, the proposed AU_GCN_CUR model demonstrated superior performance, attaining accuracies of 84.80% and 80.40% in *k*-fold and LOSO evaluations, respectively, significantly outperforming the baseline models and highlighting its effectiveness in micro-expression recognition tasks.

The performance gains observed with the 3D-CNN model suggest that, when further combined with the GCN module, the system can effectively learn the intricate relationships between facial muscle activations and various micro-expression categories. This leads to a deeper and more accurate understanding of subtle facial behaviors.

As shown in Table 6, among several micro-expression recognition models, the proposed AU_GCN_CUR demonstrates the best overall performance. On the CASME II dataset, handcrafted methods like LBP-TOP achieve moderate F1-scores but lack temporal modeling. Deep-learning baselines, such as CNN+LSTM, show higher accuracy yet an unbalanced F1. Our *AU_GCN_CUR* outperforms all, with 84.87% accuracy and 77.93% F1, due to asymmetric AU graphs and emotion modulation (e.g., LBP-TOP [11], MDMD [44]) as well as most deep learning approaches (e.g., CNN+LSTM [32], CapsuleNet [34], MER-GCN [33]).

Among these methods, LBP-TOP—recognized as one of the stronger handcrafted baselines—yields an F1-score of 42.4%, which is slightly lower than that of our model. While the CNN+LSTM model achieves a relatively high accuracy of 60.98%, its F1-score remains inferior to that of AU_GCN_CUR, indicating less balanced classification performance.

Overall, the results confirm that AU_GCN_CUR not only delivers superior classification accuracy but also excels in terms of balanced performance as reflected by the F1-score. This makes it an effective and robust solution for micro-expression recognition tasks.

### 4.2. User Subjective Perception Study

To further evaluate the practical impact of micro-expressions on emotional expressiveness in virtual humans, we designed a user perception experiment combining visual stimuli and subjective questionnaires.

The experiment employed system-generated 3D virtual human animation videos as stimulus materials, covering the six basic emotion categories proposed by Ekman: anger, disgust, fear, happiness, sadness, and surprise. Two types of videos were used: Video A presented basic emotional expressions without micro-expressions, while Video B included micro-expressions integrated into the same basic emotional expressions.

#### 4.2.1. Participant Demographics

A total of 82 participants were recruited for the experiment, including 37 males (45.1%) and 45 females (54.9%). Participants ranged in age from 18 to over 51 years, with the majority (50.0%) falling within the 25–30 age group. In terms of educational background, over 90% held a bachelor’s degree or higher, and 39.0% possessed a master’s degree or above.

All participants provided informed consent prior to the experiment, acknowledging that their data would be anonymized and used solely for academic research purposes. The entire experimental procedure was approved by an institutional ethics committee to ensure compliance with ethical standards for research involving human subjects.

#### 4.2.2. Experimental Hypothesis and Questionnaire Design

The proposed null hypothesis states that there is no significant difference between Video A and Video B in terms of emotional clarity, naturalness, and authenticity.

For each pair of A/B videos within a given emotion category, participants were asked to independently rate the two videos across the three dimensions using a 5-point Likert scale (where 1 indicates “very low” and 5 indicates “very high”). This design allows us to quantitatively assess the impact of micro-expressions on user perception.

### 4.3. Data Analysis

**Figure 5:**
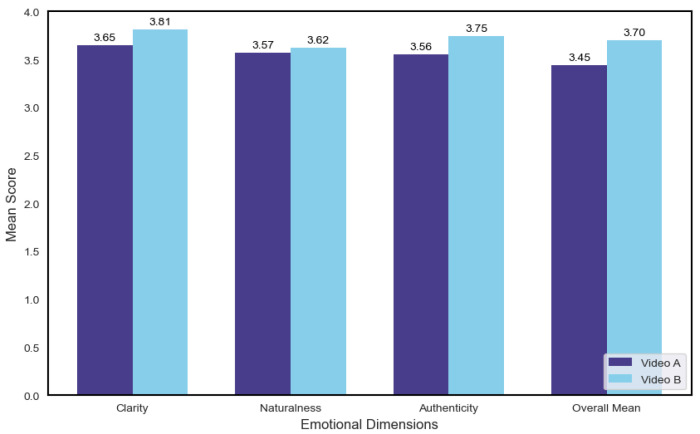
Multivariate Analysis of Variance (MANOVA) Results for Emotional Dimensions.

A Multivariate Analysis of Variance (MANOVA) was conducted to examine the overall effect of video type (A vs. B) on the three subjective rating dimensions. The results indicated that individual differences in clarity (p=0.137), naturalness (p=0.606), and authenticity (p=0.070) did not reach statistical significance. However, the overall mean score (All_Mean) for Video B was significantly higher than that for Video A (p=0.005), as shown in Figure 5.

These findings suggest that the inclusion of micro-expressions in virtual human animations leads to higher overall user approval, even if differences in individual perceptual dimensions are not independently significant.

To further investigate the effect of micro-expressions on specific emotions, separate MANOVA tests were conducted for each of the six basic emotions. The results are summarized in Table 7.

As shown, the emotion category of fear received significantly higher ratings in Video B compared to Video A (p=0.013), while disgust approached the threshold of significance (p=0.096). Other emotions, such as happiness and surprise, did not exhibit notable differences between the two video types.

**Figure 6:**
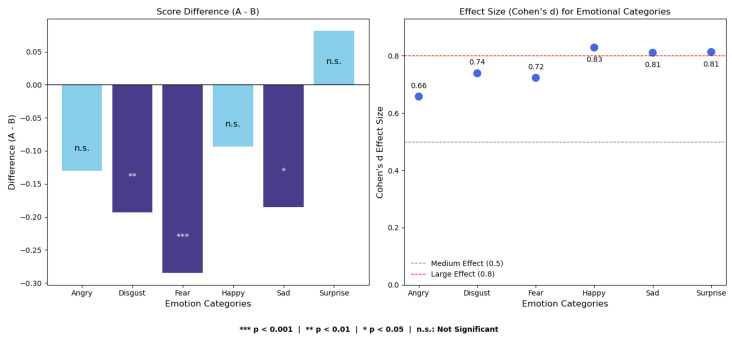
Paired sample *t*-test results for emotion-specific comparisons. Only fear and disgust remained statistically significant after Bonferroni correction (α=0.0083).

As shown in Table 8, paired sample *t*-tests were conducted for each of the six basic emotions. The results revealed significant increases in perceived scores for fear (p<0.001) and disgust (p=0.008) in Video B, while sadness approached marginal significance (p=0.044). According to the Bonferroni correction threshold (α=0.0083), only the first two effects can be considered statistically robust. The visual results in Figure 6 further emphasize that fear and disgust were the two emotions with the most notable improvement in perceived expression quality in Video B.

Effect size analysis using Cohen’s *d* revealed values ranging from 0.658 to 0.829, indicating medium to large effects. According to Cohen’s criteria [50], d=0.2 is considered a small effect, d=0.5 is considered a medium effect, and d=0.8 or above is a large effect. The value d=0.723 observed in our study falls within the medium range; however, in the context of micro-expression recognition—where perceptual signals are subtle and subjective noise is high—such an effect size is considered practically meaningful. In particular, even slight improvements in clarity or authenticity may significantly impact user experience and emotional understanding in real-world human–computer interaction scenarios.

To assess the consistency of participants’ subjective evaluations, additional paired sample *t*-tests were conducted on the three perceptual dimensions: clarity, naturalness, and authenticity. The results are presented in Table 9.

Significant improvements were observed in both clarity (p<0.001) and authenticity (p=0.004) following the inclusion of micro-expressions, while no significant difference was found for naturalness (p=0.460).

It is worth noting that although Video B received slightly higher ratings than Video A in the naturalness dimension, the difference did not reach statistical significance (p=0.460). This outcome may be attributed to the inherently short duration and subtle intensity of micro-expressions, which may not be sufficient to noticeably influence the overall smoothness and coordination of facial movements.

**Figure 7:**
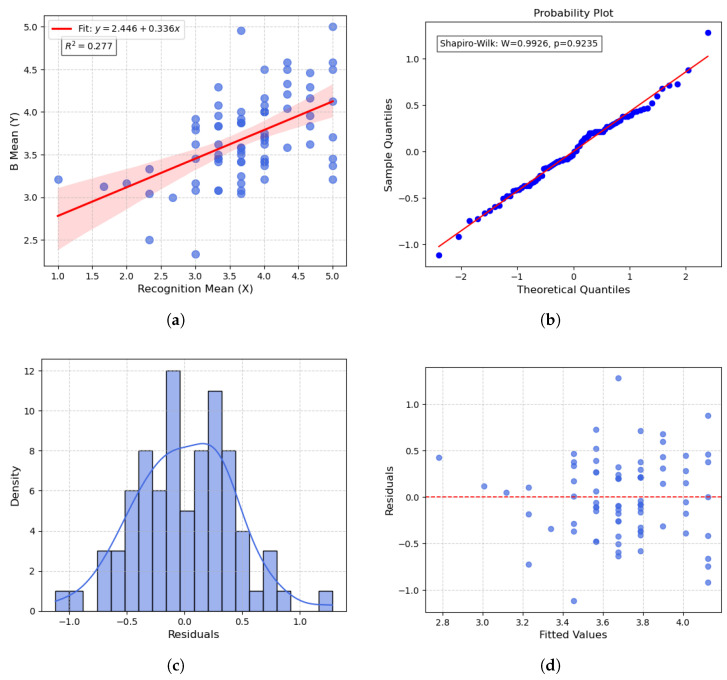
Regression Analysis and Residual Diagnostics. Each subfigure corresponds to: (**a**) Regression Model with Fitted Line; (**b**) Q-Q Plot for Residuals; (**c**) Residual Histogram with KDE Curve; (**d**) Residuals vs. Fitted Values.

Finally, a regression analysis was conducted to further examine the predictive effect of the “virtual human recognition score” on the emotional expression ratings of Video B. As shown in Figure 7, the results indicate a significant positive correlation between the two variables ($R^{2}=0.277$, p<0.001), satisfying key regression assumptions including normality and independence of residuals.

Detailed regression parameters are reported in Table 10.

### 4.4. Questionnaire Analysis Results

Based on the experimental data and statistical analyses, the following key findings can be summarized:

1. In terms of overall perceptual ratings, Video B was rated significantly higher than Video A, indicating that the inclusion of micro-expressions had a positive impact on the overall user experience.
2. In the analysis of the six basic emotions, Video B showed significantly higher ratings for fear (p<0.001, d=0.723) and disgust (p=0.008, d=0.739), both of which met the Bonferroni-corrected significance threshold (α=0.0083). This suggests that micro-expressions notably enhanced the expressiveness and perceptual salience of specific negative emotions in virtual humans.
3. In the paired sample *t*-tests, Video B also received significantly higher ratings than Video A in the dimensions of clarity (p<0.001, d=0.678) and authenticity (p=0.004, d=0.686), indicating that micro-expressions improved both the detail and credibility of facial expressions.
4. Regression analysis further revealed that participants’ recognition scores of the virtual human significantly predicted their ratings of emotional expressiveness ($R^{2}=0.277$, p<0.001). The regression model met the assumptions of residual normality and independence, indicating a strong positive correlation between recognition clarity and emotion perception.

In summary, the inclusion of micro-expressions not only enhanced the perceived realism and recognizability of emotional expressions—particularly in high-discrimination categories such as *fear* and *disgust*—but also underscored the importance of high-fidelity emotional expression in virtual human interaction.

## 5. Conclusions

This paper proposes an integrated framework for micro-expression recognition and 3D animation generation in virtual human systems, constructing a closed-loop pipeline that encompasses recognition, extraction, reconstruction, and animation driving. Spatiotemporal joint modeling is achieved through 3D-ResNet-18, while a co-occurrence-based graph convolutional network (GCN) captures structural dependencies among facial action units (AUs), enhancing the accuracy of micro-expression temporal localization and semantic representation consistency. The recognition results are mapped to animation curves, driving facial expressions in virtual humans to achieve fine-grained, realistic emotional dynamics rendering in 3D space.

Objective evaluations demonstrate that the proposed AU_GCN_CUR model outperforms multiple baselines on the CASME II dataset, achieving an F1-score of 42.93%, confirming its effectiveness and robustness in micro-expression recognition tasks. Subjective experiments indicate that incorporating micro-expressions significantly improves user ratings for clarity and authenticity, particularly for negative emotions such as fear and disgust, with notable enhancements in discriminability and emotional conveyance performance.

In summary, this study bridges micro-expression recognition and animation control, providing a structured and controllable solution for high-fidelity emotional modeling in virtual humans. Future work will integrate Transformer decoders and diffusion generative models to explore efficient and realistic micro-expression strategies, and incorporate Unity or Unreal Engine to enhance the naturalness and credibility of emotional human–machine interactions.

## Figures and Tables

**Table 1:** Comparison of representative methods for micro-expression recognition and facial animation.

Method	Category	Key Features	Strengths	Limitations
LBP-TOP [11]	Handcrafted	Local binary patterns on three orthogonal planes	Robust to illumination; low computational cost	Limited to static features; poor temporal dynamics
HOOF [12]	Handcrafted	Histogram of oriented optical flow	Captures motion cues effectively	Sensitive to noise; no AU interdependencies
CNN + LSTM [32]	Deep Learning	Spatial CNN with temporal LSTM	Models sequential dependencies	Ignores AU structural relationships; high complexity
MER-GCN [33]	Deep Learning	Graph convolutional network for MER	Captures AU co-occurrences	Symmetric graphs only; no emotion modulation
CapsuleNet [34]	Deep Learning	Capsule networks for feature routing	Handles part-whole relationships	Limited temporal integration; no animation mapping
SARA [29]	Animation System	Behavior Markup Language (BML)	Supports basic emotion synthesis	Rigid transitions; no micro-expression support
ARIA [30]	Animation System	Modular input–processing–output	Flexible architecture	Relies on predefined templates; low granularity
AU_GCN _CUR	Deep Learning + Graph	3D-ResNet + Symmetric GCN	Joint spatiotemporal-AU modeling; end-to-end animation	Higher computational load for graphs

**Table 2:** Architecture of the 3D-ResNet-18 network. The input is organized as (T,H,W), where *T* denotes the temporal frame length, and *H* and *W* represent the height and width of the input image, respectively. Each residual block specifies the kernel size, number of channels, and downsampling strategy.

Layer	Kernel Size	Output Size
Conv1	3×7×7,64, stride 1×2×2	T×56×56
ResBlock1	3×3×3,64 3×3×3,64×2	T×56×56
ResBlock2	3×3×3,128 3×3×3,128×2	$\frac{T}{2}\times 28\times 28$
ResBlock3	3×3×3,256 3×3×3,256×2	$\frac{T}{4}\times 14\times 14$
ResBlock4	3×3×3,512 3×3×3,512×2	$\frac{T}{8}\times 7\times 7$
Global Avg. Pooling	Spatial-temporal pooling	512×1

**Table 3:** Representative AU-to-rig control mapping used to drive curves in the Unreal-based facial rig. Control names keep the original left/right channel naming.

AU	FACS Description	Rig Control Channels (L/R as Named in the Rig)
AU1	Inner brow raiser	CTRL_L_brow_raiseIn; CTRL_R_brow_raiseIn
AU2	Outer brow raiser	CTRL_L_brow_raiseOut; CTRL_R_brow_raiseOut
AU4	Brow lowerer	CTRL_L_brow_down; CTRL_R_brow_down; CTRL_L_brow_lateral; CTRL_R_brow_lateral
AU5	Upper lid raiser	CTRL_L_eye_eyelidU; CTRL_R_eye_eyelidU
AU6	Cheek raiser	CTRL_L_eye_cheekRaise; CTRL_R_eye_cheekRaise
AU7	Lid tightener	CTRL_L_eye_squintInner; CTRL_R_eye_squintInner
AU9	Nose wrinkler	CTRL_L_nose; CTRL_R_nose; CTRL_R_nose_wrinkleUpper; CTRL_L_nose_wrinkleUpper
AU10	Upper lip raiser	CTRL_L_mouth_upperLipRaise; CTRL_R_mouth_upperLipRaise
AU12	Lip corner puller	CTRL_L_mouth_cornerPull; CTRL_R_mouth_cornerPull
AU15	Lip corner depressor	CTRL_L_mouth_cornerDepress; CTRL_R_mouth_cornerDepress
AU20	Lip stretcher	CTRL_L_mouth_stretch; CTRL_R_mouth_stretch
AU23	Lip tightener	CTRL_L_mouth_tightenU; CTRL_R_mouth_tightenU; CTRL_L_mouth_tightenD; CTRL_R_mouth_tightenD

**Table 4:** Accuracy and standard deviation of baseline and proposed models under *k*-fold and LOSO evaluation.

Model	*k*-fold Acc (%)	LOSO Acc (%)	Code Rerun
3D-ResNet (pre-trained)	61.92 (±6.53)	56.20 (±28.31)	Yes
3D-ResNet	84.87 (±3.80)	80.51 (±6.58)	Yes
AU_GCN_CUR	84.80 (±3.89)	80.40 (±6.13)	Yes

**Table 5:** Comparison of accuracy (%) under *k*-fold and LOSO evaluation.

Method	*k*-fold Accuracy (%)	LOSO Accuracy (%)
ThreeDFlow	63.48	42.58
CNN + LSTM	63.78	48.35
CapsuleNet	44.83	31.16
**AU_GCN_CUR**	**84.80**	**80.40**

Note: Bold values indicate the best performance achieved by the proposed AU_GCN_CUR method.

**Table 6:** Micro-Expression Recognition Results on the CASME II Dataset.

Category	Method	Accuracy (%)	F1-Score (%)
Hand-crafted	MDMD [44]	57.07	23.50
	SP-FD [45]	21.31	12.43
	OF-FD [46]	37.82	35.34
	LBP-TOP [11]	56.98	42.40
	LOCP-TOP [16]	45.53	42.25
Deep-learning	MER–GCN [33]	54.40	30.30
	SOFTNe [47]	24.10	20.22
	Concat–CNN [48]	25.05	20.19
	LSSNet [49]	37.70	32.50
	AU_GCN_CUR *	**84.87**	**77.93**

Note: Previous works on CASME II typically only report mean accuracy/F1 without standard deviation. The algorithm marked with an asterisk represents our proposed method, with results derived from our own experimental evaluations. The others are benchmarks from prior studies, all conducted on the same CASME II dataset. Bold values indicate the best performance achieved by the proposed AU_GCN_CUR method.

**Table 7:** MANOVA Results for Emotion-Specific Differences between Video A and Video B.

Emotion	Mean A	Mean B	Diff (A-B)	*p*-Value	Cohen’s *d*
Anger	3.6931	3.8232	−0.1301	0.207	0.658
Disgust	3.6362	3.8293	−0.1931	0.096	0.739
Fear	3.5305	3.8150	−0.2846	0.013	0.723
Happiness	3.4939	3.5874	−0.0935	0.471	0.829
Sadness	3.5528	3.7378	−0.1850	0.146	0.811
Surprise	3.6667	3.5854	0.0813	0.523	0.813
Overall	3.4463	3.7027	−0.2564	0.005	0.048

**Table 8:** Paired Sample *t*-Tests Comparing Perceived Expression Quality Between Video A and Video B for Each Basic Emotion.

Dimension	Mean A	Mean B	Diff (A-B)	*p*-Value	Cohen’s *d*
Anger	3.6931	3.8232	−0.1301	0.068	0.658
Disgust	3.6362	3.8293	−0.1931	**0.008 **	0.739
Fear	3.5305	3.8150	−0.2846	**<0.001**	0.723
Happiness	3.4939	3.5874	−0.0935	0.325	0.829
Sadness	3.5528	3.7378	−0.1850	**0.044**	0.811
Surprise	3.6667	3.5854	0.0813	0.425	0.813

Note: Bold *p*-values indicate statistical significance at the 0.05 level.

**Table 9:** Clarity, Naturalness, and Authenticity Paired Sample *t*-Tests.

Dimension	Mean A	Mean B	Diff (A-B)	*p*-Value	Cohen’s *d*
Clarity	3.6545	3.8130	−0.1585	**<0.001**	0.678
Naturalness	3.5730	3.6220	−0.0488	0.460	0.595
Authenticity	3.5600	3.7500	−0.1950	**0.004**	0.686

Note: Bold *p*-values indicate statistical significance at the 0.05 level.

**Table 10:** Regression Analysis: Predicting Perceived Emotional Expression in Video B.

Variable	Coefficient	Std. Error	*t*-Value	*p*-Value
Intercept (Constant)	2.446	0.232	10.558	**<0.001**
Recognition_Mean	0.336	0.061	5.542	**<0.001**
Model Summary	R^2^ = 0.277	Adj. R^2^ = 0.268	F = 30.72	***p*** **< 0.001**
Shapiro-Wilk Test	W = 0.9926	*p* = 0.9235	(Residuals are normally distributed)	

Note: Bold *p*-values indicate statistical significance at the 0.05 level.

## Data Availability

The original contributions presented in this study are included in the article. Further inquiries can be directed to the corresponding author.

## Supplementary Materials

The following supporting information can be downloaded at: https://www.mdpi.com/article/10.3390/biomimetics10090587/s1, Table S1: Mapping of Action Units (AUs) to 3D control curve names for facial animation.
