# Supplementary material for: Graph-Driven Micro-Expression Rendering with Emotionally Diverse Expressions for Lifelike Digital Humans
Source: Biomimetics (Basel). 2025 Sep 3;10(9):587. doi: 10.3390/biomimetics10090587 (PMC12467307; doi:10.3390/biomimetics10090587)

Supplementary Material – AU Comparison Table

Action Unit	Description	Control Curves	Image
AU1	inner brow raiser	CTRL_L_brow_raiseIn<br>CTRL_R_brow_raiseIn	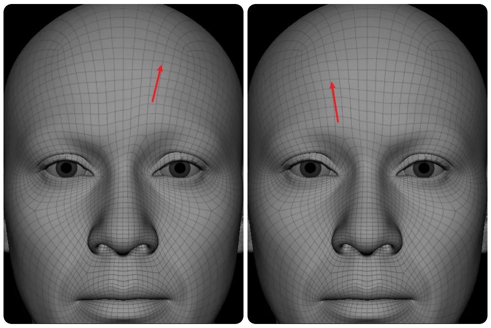
AU2	outer brow raiser	CTRL_L_brow_raiseOut<br>CTRL_R_brow_raiseOut	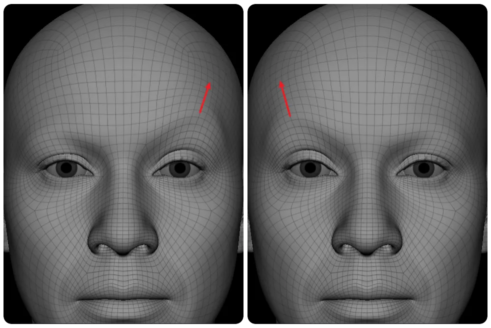
AU4	brow lowerer	CTRL_L_brow_down<br>CTRL_R_brow_down<br>CTRL_L_brow_lateral<br>CTRL_R_brow_lateral	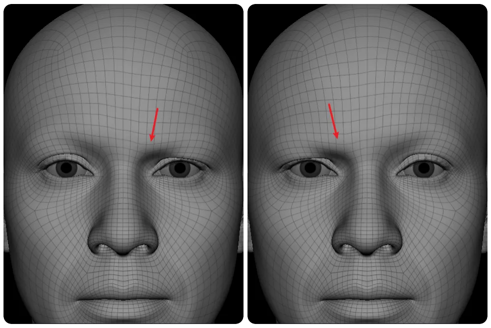<br>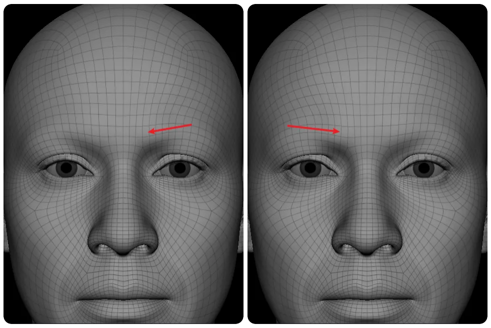

AU5	upper lid raiser	CTRL_L_eye_eyelidU<br>CTRL_R_eye_eyelidU	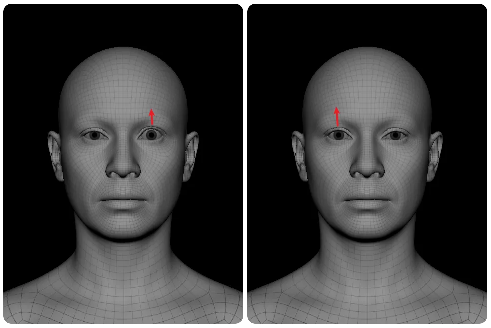
AU6	cheek raiser	CTRL_L_eye_cheekRaise<br>CTRL_R_eye_cheekRaise	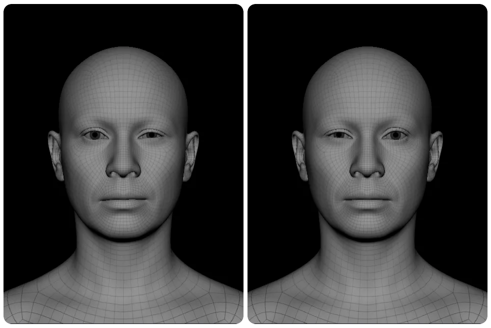
AU7	lid tightener	CTRL_L_eye_squintInner<br>CTRL_R_eye_squintInner	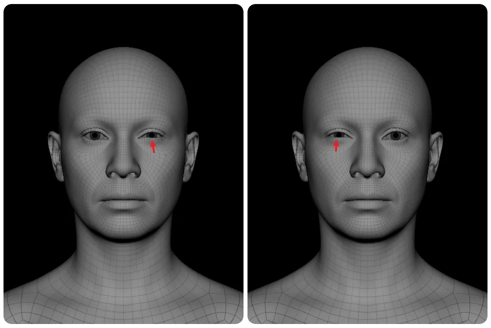
AU9	nose wrinkler	CTRL_L_nose<br>CTRL_R_nose<br>CTRL_R_nose_wrinkleUpper<br>CTRL_L_nose_wrinkleUpper	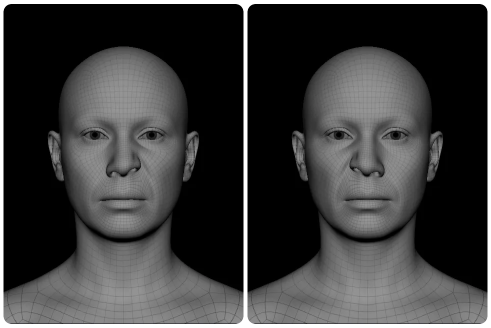
AU10	upper lip raiser	CTRL_L_mouth_upperLipRaise<br>CTRL_R_mouth_upperLipRaise	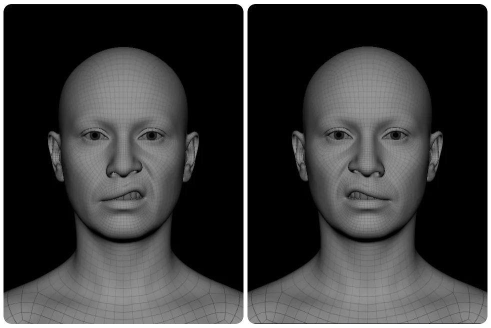

AU11	nasolabial<br>furrow<br>deepener	CTRL_L_nose_nasolabialDeepen<br>CTRL_R_nose_nasolabialDeepen	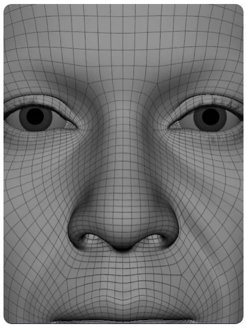 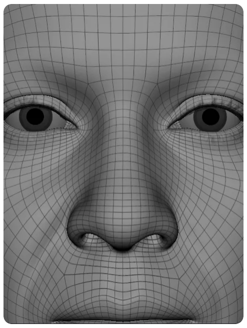
AU12	lip corner puller	CTRL_L_mouth_cornerPull<br>CTRL_R_mouth_cornerPull	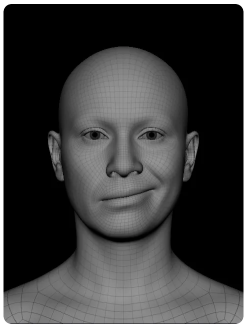 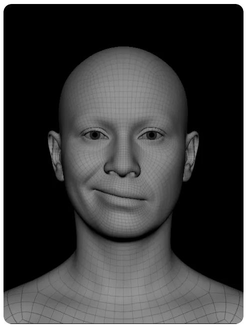
AU13	sharp lip puller	CTRL_L_mouth_sharpCornerPull<br>CTRL_R_mouth_sharpCornerPull	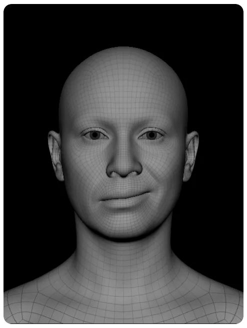 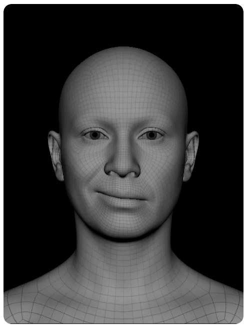
AU14	dimpler	CTRL_L_mouth_dimple<br>CTRL_R_mouth_dimple	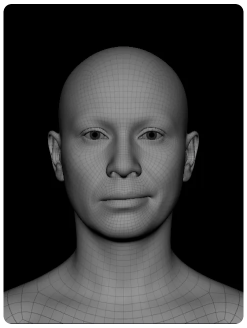 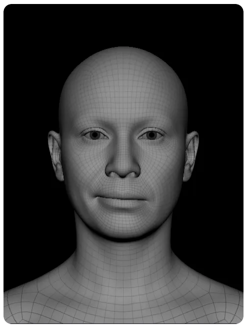
AU15	lip corner<br>depressor	CTRL_L_mouth_cornerDepress<br>CTRL_R_mouth_cornerDepress	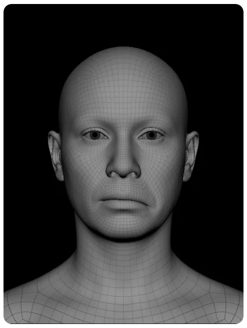 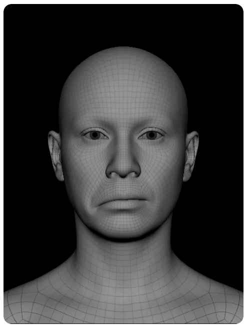

AU16	lower lip depressor	CTRL_L_mouth_lowerLipDepress<br>CTRL_R_mouth_lowerLipDepress	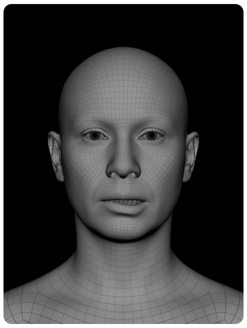 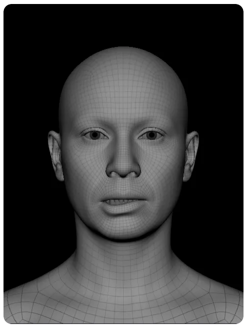
AU17	chin raiser	CTRL_L_jaw_ChinRaiseD<br>CTRL_R_jaw_ChinRaiseD	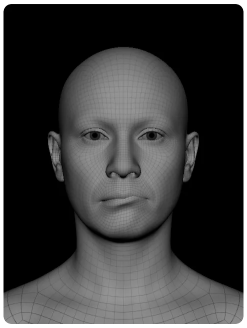 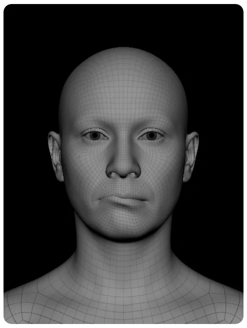

AU18	lip pucker	CTRL_L_mouth_purseU<br>CTRL_R_mouth_purseU<br>CTRL_L_mouth_purseD<br>CTRL_R_mouth_purseD<br>CTRL_L_mouth_lipsTowardsTeethU<br>CTRL_R_mouth_lipsTowardsTeethU<br>CTRL_L_mouth_lipsTowardsTeethD<br>CTRL_R_mouth_lipsTowardsTeethD	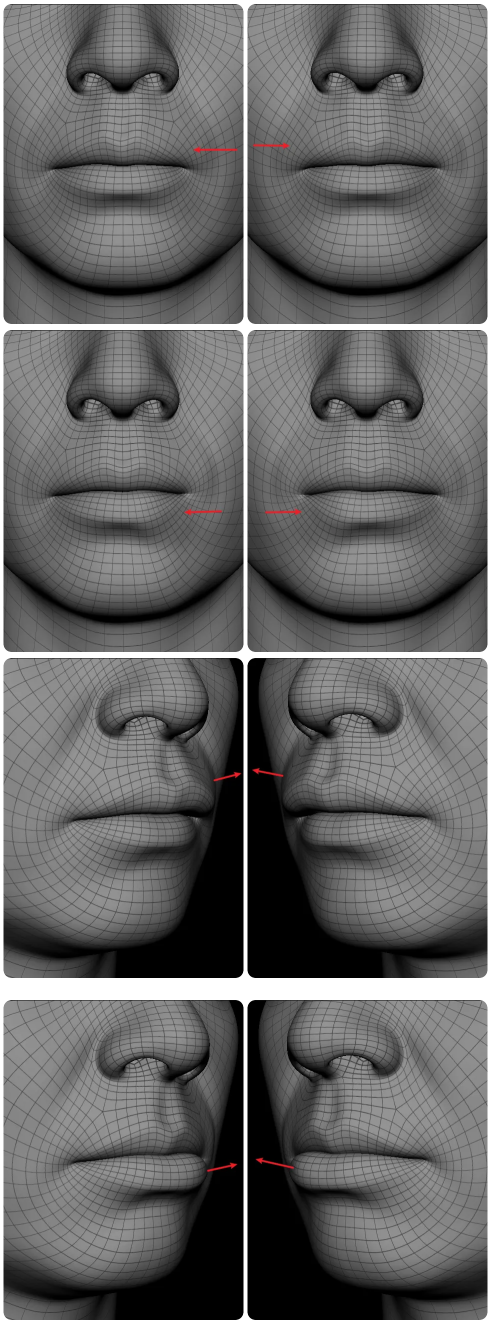
AU20	lip stretcher	CTRL_L_mouth_stretch<br>CTRL_R_mouth_stretch	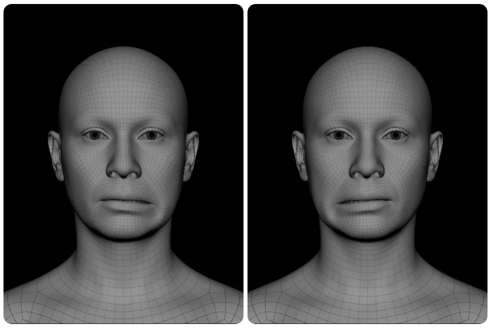

AU22	lip funneler	CTRL_L_mouth_funnelU<br>CTRL_R_mouth_funnelU<br>CTRL_L_mouth_funnelD<br>CTRL_R_mouth_funnelD	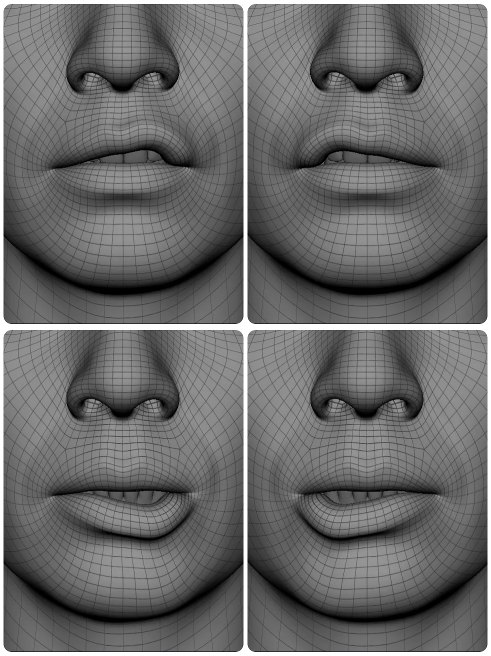
AU23	lip tightener	CTRL_L_mouth_tightenU<br>CTRL_R_mouth_tightenU<br>CTRL_L_mouth_tightenD<br>CTRL_R_mouth_tightenD	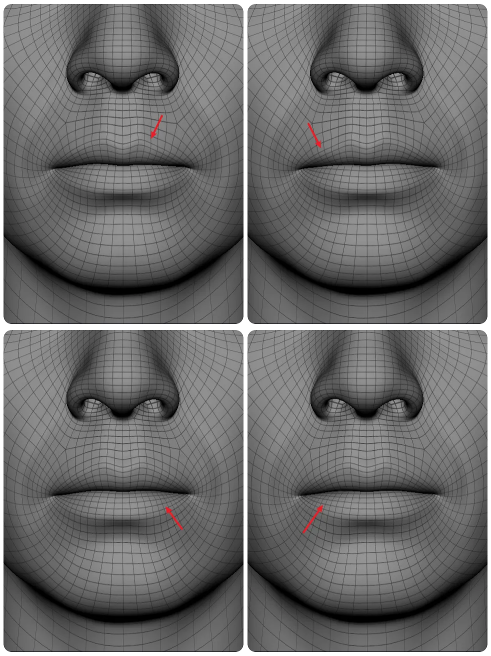
AU27	mouth stretch	CTRL_C_jaw	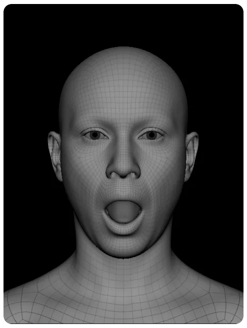

AU28	lip presser	CTRL_L_mouth_lipsPressU<br>CTRL_R_mouth_lipsPressU<br>CTRL_L_mouth_lipsPressD<br>CTRL_R_mouth_lipsPressD	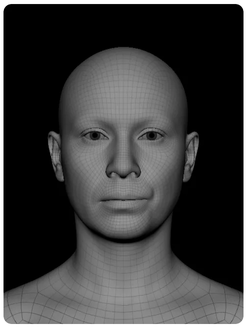 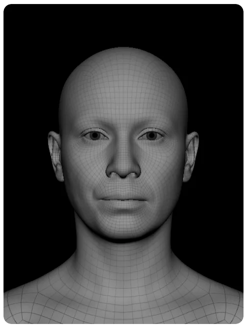
AU29	jaw thurst	CTRL_C_jaw_fwdBack	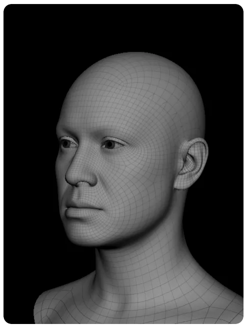
AU34	puff	CTRL_L_mouth_suckBlow<br>CTRL_R_mouth_suckBlow	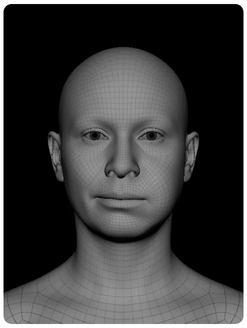 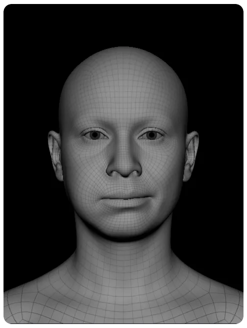
AU43	eye closure	CTRL_R_eye_blink<br>CTRL_L_eye_blink	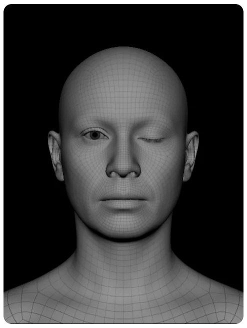 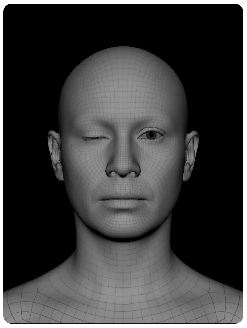
AU45	blink		
AU63	eyes up	CTRL_L_eye<br>CTRL_R_eye	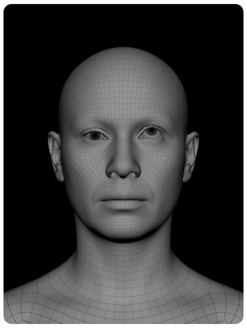 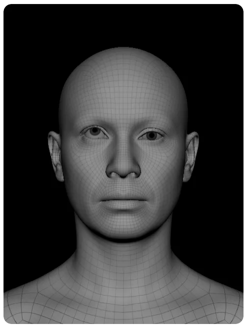

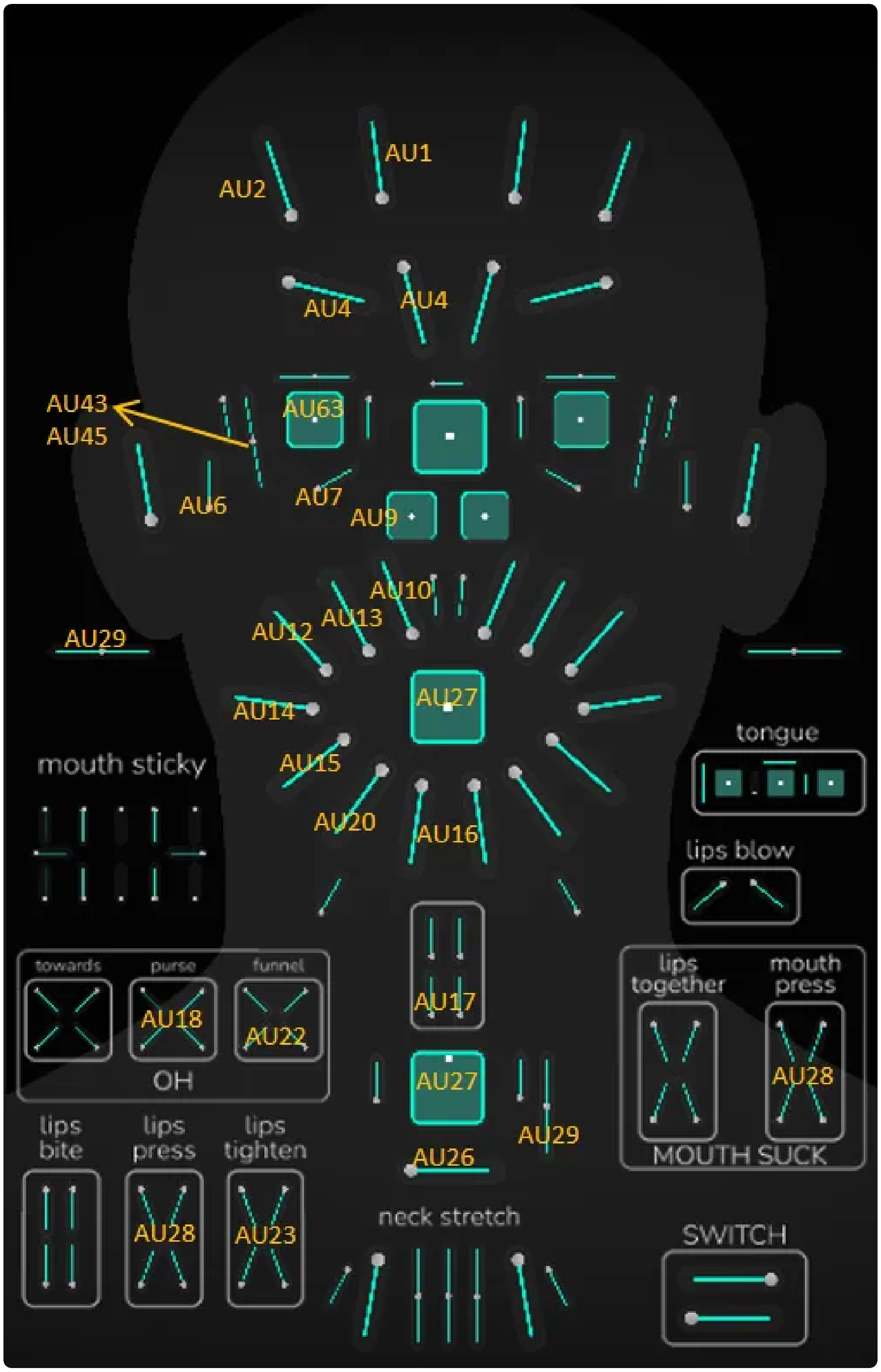
AU1
AU2
AU4
AU4
AU43
AU45
AU63
AU6
AU7
AU9
AU10
AU13
AU12
AU29
AU14
AU27
AU15
AU20
AU16
mouth sticky
tongue
lips blow
towards
purse
funnel
AU18
AU22
OH
AU17
lips together
mouth press
AU27
AU29
AU28
MOUTH SUCK
lips bite
lips press
lips tighten
AU26
AU28
AU23
neck stretch
SWITCH

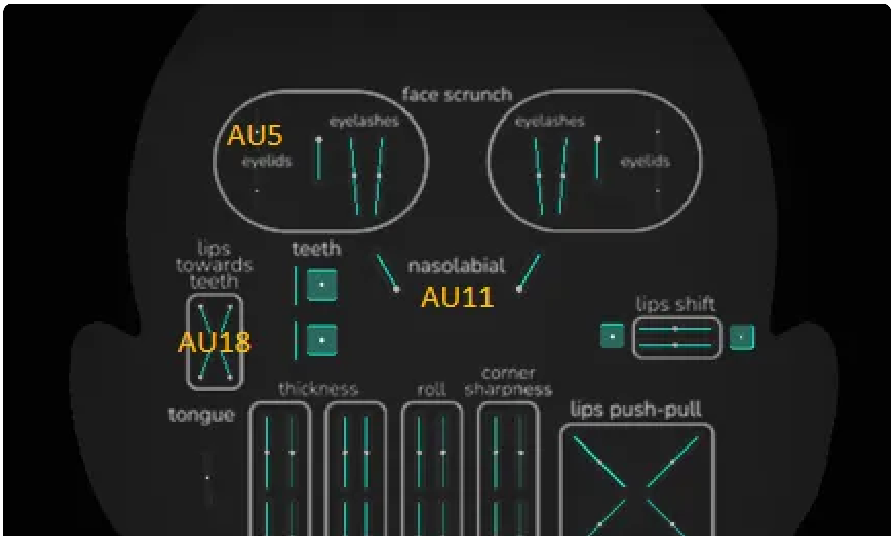
face scrunch
AU5
eyelashes
eyelids
eyelashes
eyelids
lips towards teeth
teeth
nasolabial
AU11
lips shift
AU18
tongue
thickness
roll
corner sharpness
lips push-pull

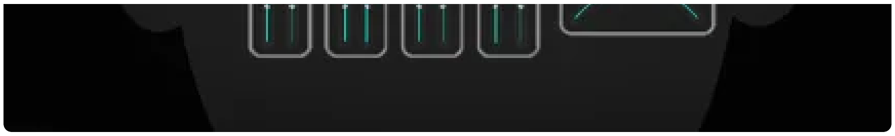

Supplement: Supplementary file 1 [file biomimetics-10-00587-s001.zip › biomimetics-3818572-supplementary.pdf]
